# Bcl-2 proteins bid and bax form a network to permeabilize the mitochondria at the onset of apoptosis

**DOI:** 10.1038/cddis.2016.320

**Published:** 2016-10-20

**Authors:** Robert F Gahl, Pallavi Dwivedi, Nico Tjandra

**Affiliations:** 1 Laboratory of Molecular Biophysics, Biochemistry and Biophysics Center, National Heart, Lung and Blood Institute, National Institutes of Health, Bethesda 20892, MD, USA

## Abstract

The most critical step in the initiation of apoptosis is the activation of the Bcl-2 family of proteins to oligomerize and permeabilize the outer-mitochondrial membrane (OMM). As this step results in the irreversible release of factors that enhance cellular degradation, it is the point of no return in programmed cell death and would be an ideal therapeutic target. However, the arrangement of the Bcl-2 proteins in the OMM during permeabilization still remains unknown. It is also unclear whether the Bcl-2 protein, Bid, directly participates in the formation of the oligomers in live cells, even though it is cleaved and translocates to the OMM at the initiation of apoptosis. Therefore, we utilized confocal microscopy to measure Förster resonance energy transfer (FRET) efficiencies in live cells to determine the conformation(s) and intermolecular contacts of Bid within these Bcl-2 oligomers. We found that Bid adopts an extended conformation, which appears to be critical for its association with the mitochondrial membrane. This conformation is also important for intermolecular contacts within the Bid oligomer. More importantly for the first time, direct intermolecular contacts between Bid and Bax were observed, thereby, confirming Bid as a key component of these oligomers. Furthermore, the observed FRET efficiencies allowed us to propose an oligomeric arrangement of Bid, Bax, and possibly other members of the Bcl-2 family of proteins that form a self-propagating network that permeabilizes the OMM.

The commitment of a cell to undergo apoptosis involves the activation and suppression of certain members of the Bcl-2 family of proteins.^1, 2^ Pro-apoptotic members such as Bax, Bak, and Bok, are activated while pro-survival members, such as Bcl-2, Bcl-x_L_, and Bcl-w, are inhibited. The culmination of interactions of the pro-apoptotic members results in the permeabilization of the outer-mitochondrial membrane (OMM). Caspases are then activated and other proteins are released which enhance apoptosis. Concurrent with these cellular events, there is a third class of member proteins of the Bcl-2 family known as the 'initiators',^3^ which undergo marked changes in cellular location. There still remain unanswered questions about what role initiators play in the regulation of apoptosis. This study focuses on identifying the conformational changes and intermolecular contacts of the initiator, Bid, in order to gain a more detailed picture of the architecture of the Bcl-2 proteins during the permeabilization of the OMM.

Bid is an alpha-helical, 22-kDa protein that adopts a characteristic *α*-helical fold shared by other members of the Bcl-2 family of proteins.^4, 5^ Bid, in a similar fashion to the pro-apoptotic member, Bax, translocates from the cytosol to the mitochondria at the initiation of apoptosis.^6, 7^ In contrast to Bax, which translocates as a full-length protein, the translocation of Bid is believed to be initiated via its cleavage at residue Leu59 by caspase-8 to produce a 7 kDa fragment, p7, and a 15 kDa fragment, p15.^7^ The p15 fragment, referred to as tBid, associates with mitochondria outer membrane (MOM) and subsequently initiating the process of its permeabilization (MOMP). This fragmentation is necessary for oligomer formation in the presence of Bax or Bak and the eventual release of cytochrome c.^8^ The cellular and molecular timelines of Bid's cleavage into p7 and tBid as well as its translocation to the mitochondrial outer membrane following apoptosis induction have been studied in the past.^9, 10, 11^ A more refined breakdown of this timeline would be very useful. A better understanding of this process might also lead to the potential development of better therapeutics that can specifically target different functional aspects of Bid.^12, 13^

Previous investigations of the initiators have focused on their unique structural features and similarities with the other Bcl-2 proteins. Some member proteins that are pro-apoptotic and pro-survival contain four homologous domains, BH1-4.^3^ The initiators, however, contain only one of these domains, BH3. These proteins are known as BH3-only proteins. Other proteins such as p53^14^ and PUMA^15^ could also act as initiators. This has led to the hypothesis that the initiators, which include Bid, utilize the BH3 domain to catalyze pro-apoptotic activity by binding to other Bcl-2 proteins. The initiators can accomplish this role by adopting the characteristic *α*-helical fold of Bcl-2 proteins like Bid or are intrinsically unfolded.^16^ Indeed, peptides from the BH3 domains of the initiators are known to bind with the pro-apoptotic members^16^ or pro-survival members^17^ to enhance pro-apoptotic activity in cell-free assays. Bid can also occupy solution and membrane environments^18^ to enhance the insertion of Bax into membranes.^19, 20^ Similar activation has been observed with other initiators in the Bcl-2 family.^21^ Although the activity of Bid has been well-studied, the possible contributions of other factors that affect its regulation could be overlooked. In a complex biological system, it is important to gather different viewpoints by studying it using various techniques, which individually cannot provide a complete picture. Cell-free assays, which constitute the majority of studies of Bcl-2 proteins, have been very successful in providing accurate descriptions on various states of the proteins. A number of factors but especially the complete components of the OMM, including apoptosis machinery and their physical condition (oxidation, chemical modification, composition, and so on) can modify the states of these proteins within this complex process. Although a wealth of knowledge has been gained from cell-free assays, there still remains the possibility that other features, such as conformational changes that might already be occurring before translocation or exchange equilibrium between membrane bound and cytosolic form of the protein, can be observed when experiments are performed under conditions more closely resembling the live-cell environment. As was shown previously and reproducibly, Bax adopts a different conformation in a test tube in comparison with its native environment within the cell.^22^ This allows for the equilibration of Bax between the cytosol and mitochondria before the initiation of apoptosis.^23, 24, 25^ Therefore, we have employed methodologies developed in our lab using live-cell quantitative Förster resonance energy transfer (FRET)-imaging to perform measurements in living cells undergoing apoptosis in order to reach biologically relevant conclusions. From observations of FRET using fluorophores located at positions on Bid as shown in Figure 1a & b, we were able to determine the structural contributions of Bid and tBid in the activation of Bax, as well as the Bcl-2 oligomers that permeabilize the OMM.

## Results

### Conformational rearrangements within bid as it translocates to the mitochondria

**Figure 1:**
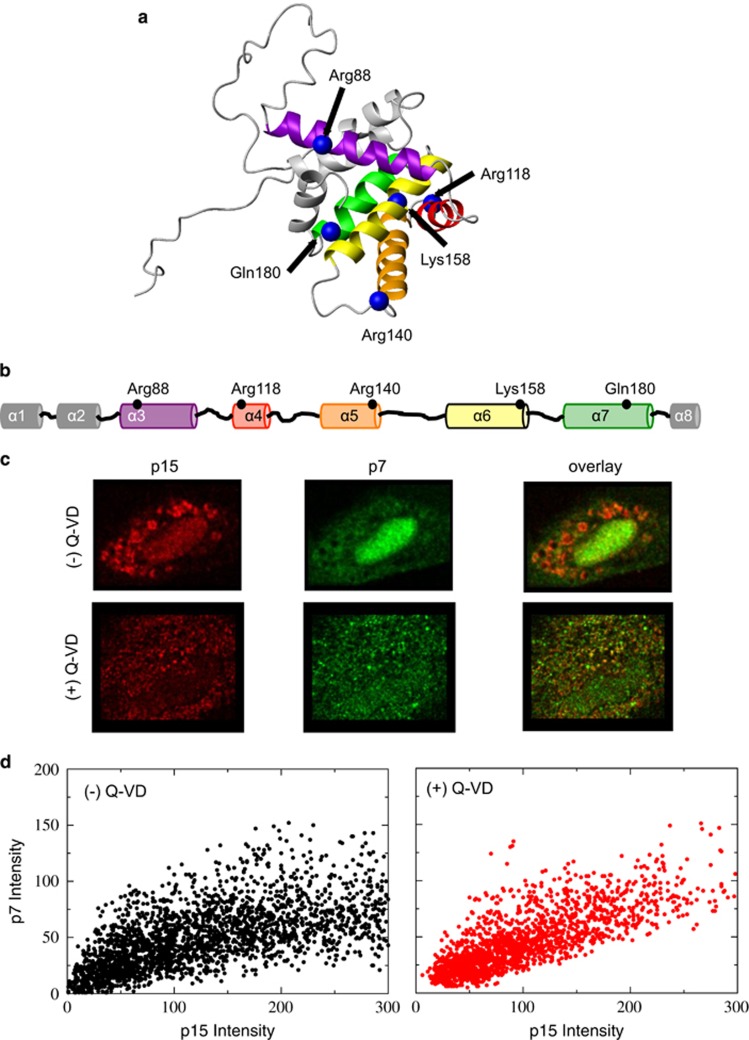
Positions to probe conformational changes and intermolecular contacts in Bid. (**a**) A ribbon representation of Bid structure (PDB ID: 2BID) shows the eight *α*-helices^5^. The C-*β* carbons of the residues that were mutated to a cysteine are shown in blue. (**b**) A linear cartoon representation of Bid shows where the probes were located in the linear sequence of Bid. The helices are color coded. A FRET donor or acceptor was attached at a particular site for the FRET experiment. (**c**) Images of AlexaFluor546-labeled p7 and AlexaFluor633-labeled p15 individually and in overlay are shown. Without Q-VD-OPh, Bid is cleaved by caspase-8 into the p7 and p15 fragments. The p15 fragment is punctate, whereas the p7 fragment is diffuse. Only p15 is translocated. The addition of the caspase inhibitor, Q-VD-OPh, allows Bid to translocate without fragmentation to p7 and p15 and (**d**) these plots show a correlation between the signals from p7 and p15 in the presence and absence of Q-VD-OPh. A correlation coefficient of 0.10 was found in the absence of Q-VD-OPh, whereas a correlation coefficient of 0.55 was found in the presence of Q-VD-OPh

We monitored the caspase activation and subsequent translocation of Bid to the mitochondria by monitoring the fluorescence intensity of AlexaFuor546 conjugated to a cysteine at position 35 (p7) relative to the intensity of AlexaFluor633 conjugated to a lysine on tBid (p15). Images of this variant after translocation are shown in Figure 1c. Consistent with the observation that the p7 and p15 fragments are formed at the initiation of apoptosis, p15 adopts a punctate distribution, whereas the p7 fragment adopts a diffuse distribution. As shown in Supplementary figure S1, the p15 fragment colocalizes with the mitochondria. The p7 fragment is free to diffuse throughout the cell after cleavage at position Leu56. However, when the caspase inhibitor Q-VD-OPh is added prior to the addition of STS, there is considerable correlation between these two fragments, Figure 1d. This demonstrates that translocation to mitochondria does not require caspase cleavage of Bid into the p7 and p15 fragment. This is consistent with a previous report that suggests that caspase-8 cleaves Bid on the mitochondria membrane and that p7 stays associated with p15 or the membrane after fragmentation.^26^

The conformational changes in the p15 fragment after translocation were probed using our optimized FRET methodology^22, 27^ and the results are shown in Table 1. A review of the theory of FRET is presented by Y Sun and colleagues.^28^ The p15 fragment adopts an extended conformation that can form intermolecular contacts, which is discussed below and in the Supplementary Information.

### Cross-linking tBid affects its translocation to the mitochondria

**Figure 2:**
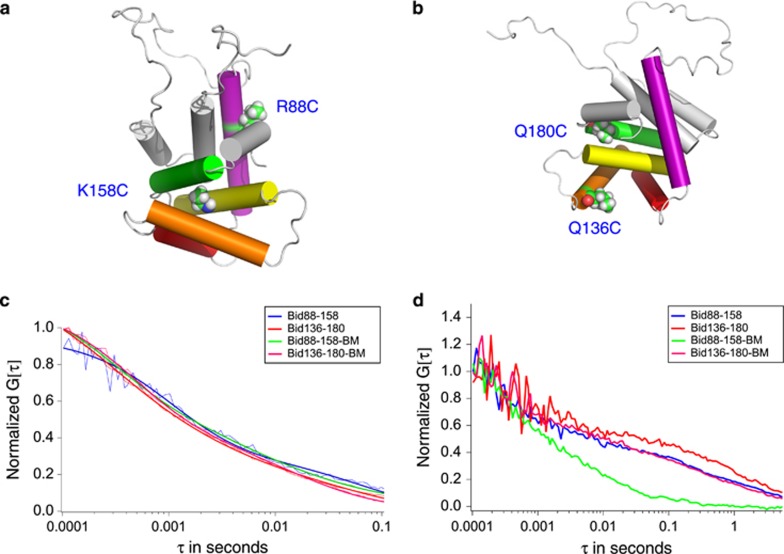
Intramolecular cross-linking affects translocation behavior. (**a**) Bid was cross-linked between positions Bid-88 and Bid-158. (**b**) Bid was also cross-linked at positions Bid-136 and Bid-180. Cylinders represent helices in the Bid structure. They are color coded as in Figure 1. (**c**) The intracellular diffusion of both cross-linked (Bid-88-158-BM in green, Bid-136-180-BM in pink) species is similar to the uncross-linked variant (Bid-88-158 in blue, Bid-136-180 in red) before translocation as determined by FCS. (**d**) After translocation, the cross-linker between positions Bid-88 and Bid-158 prevented translocation, but not the cross-linker between positions Bid-136 and Bid-180 in Bid. The same color code was used as in 2C

Bid was cross-linked at two different sets of positions shown in Figures 2a and b to test which conformational changes are necessary for translocation. These locations are not expected to affect their caspase-8 cleavage activity because the loop containing Leu56 is still exposed. Before translocation, the intracellular diffusion of the uncross-linked variants and cross-linked variants of Bid are very similar, Figure 2c. The auto-correlation function for each of the Bid variants could be fit with two diffusive behaviors that correspond to a fast and slow time constant. The majority (67–71%) of the diffusive behavior exhibited the fast time constant (7.3 × 10^−4^ to 1.6 × 10^−3^ s). The rest of the diffusive behavior (29–33%) exhibited the slow time constant (3.3 × 10^−2^ to 1.1 × 10^−1^ s). These relative populations are the result of fitting without correction to differences in quantum yields for the two time constants. As a result, they are only 'apparent populations'. In addition, it is not clear whether to fit the slower time constant to a 3-D or 2-D diffusion model because multiple scenarios could account for the different models of diffusion at a time constant of this magnitude, such as oligomers, association with an organelle or diffusion within a membrane environment. However, these apparent populations are reported to demonstrate the heterogeneity of the diffusion of Bid in a cellular environment.

After translocation, the intracellular diffusion of these variants differs significantly from that of the uncross-linked variant (Figure 2d). The uncross-linked variants of Bid diffuse at a significantly slower rate, which is consistent with the translocation of Bid to the MOM or the formation of large oligomers. However, cross-linking positions Bid-88 and Bid-158 abrogated translocation. The diffusion of Bid cross-linked at positions 88 and 158 was almost identical before and after the addition of STS. In contrast, cross-linking Bid at positions Bid-136 and Bid-180 did not affect translocation. The diffusive behavior of this variant, before and after the addition of STS, was the same with or without the cross-linker. By cross-linking positions 136 and 180, the helix that contains the BH3 helix is free to associate with the OMM. Thus, these results demonstrate that in order to associate with the OMM, Bid must adopt an extended conformation and have the BH3 domain exposed.

### Intermolecular contacts between tBid molecules at the OMM

**Figure 3:**
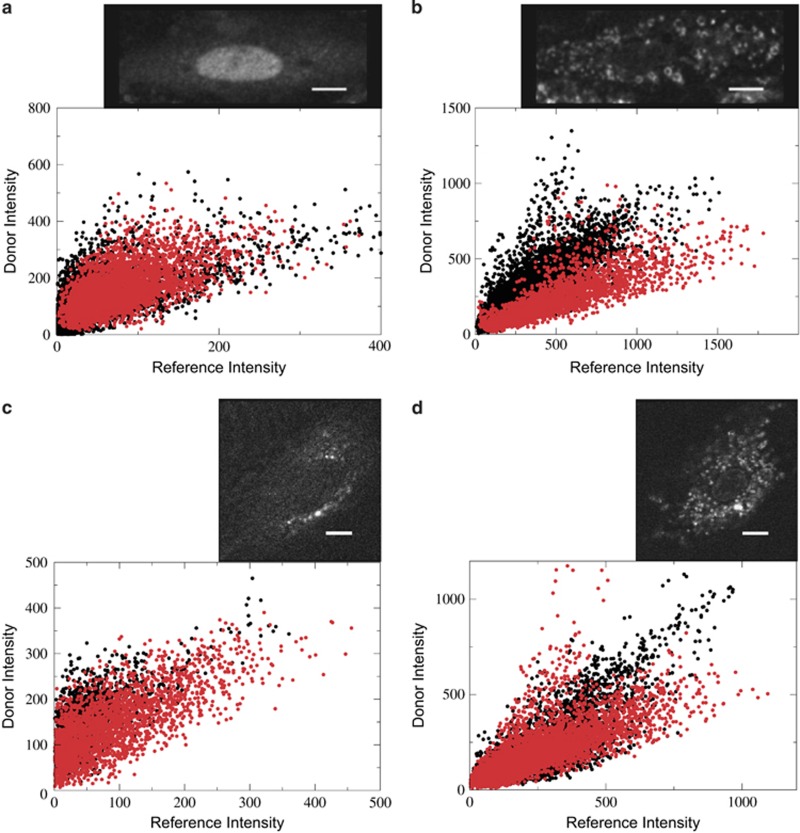
Intermolecular contacts between tBid molecules. (**a**) Before translocation, preSTS, there is no FRET between Bid-158 and Bid-118. (**b**) After translocation, postSTS, FRET is observed between the two sites as indicated by a lower correlative slope when the FRET Acceptor is present, red dots, compared with when it is absent, black dots. (**c**) There is no FRET between Bid-118 and Bid-88 before translocation. (**d**) A similar correlative trend in the presence (red dots) and absence (black dots) of the acceptor, indicates that there is no FRET between Bid-118 and Bid-88 after translocation

FRET between separate molecules of tBid at the OMM were also measured. These experiments were carried out as described before.^22^ Figure 3 contains images that show the translocation of Bid from the cytosol to the mitochondria, as well as a per-pixel scatterplot that utilizes correlative trends to determine if there is FRET between two sites within tBid, as analyzed previously.^22^ In both experiments, the intracellular distribution of Bid is diffuse and punctate before and after translocation to the mitochondria, respectively. In Figure 3a, before translocation, there is no FRET between positions Bid-158 and Bid-118 of tBid, as evidenced by the similar correlative slope in the presence and absence of the FRET acceptor. After translocation, however, in Figure 3b, the correlative slope in the presence of the acceptor is lower, which indicates FRET between positions Bid-158 and Bid-118 of tBid after translocation. In Figures 3c and d, contact between positions Bid-118 and Bid-88 of tBid were investigated. As indicated by their similar slopes in the scatter plots, there is no FRET between these sites and presumably no contact between positions Bid-118 and Bid-88.

Intermolecular FRET efficiencies between other positions are shown in Table 2. Most of the contacts between tBid molecules are between the N-terminal of one molecule, positions Bid-88 and Bid-118, and the C-terminal region of another molecule, positions Bid-158 and Bid-180. The absence of FRET between symmetric regions in tBid indicates that an extended conformation is required to satisfy these distance restraints. This is in agreement with the picture formulated from intramolecular FRET efficiencies. One of the observed contacts is between positions Bid-118 and Bid-180, which explains the residual FRET efficiency after translocation in the intramolecular FRET experiments. The magnitude of some of these FRET efficiencies is similar to those observed in the intermolecular contacts between Bax molecules reported previously.^22^ The FRET efficiencies between positions Bid-118, Bid-140, and Bid-158 are particularly interesting. The observed intermolecular FRET efficiencies between positions Bid-118 and Bid-158 and between Bid-140 and Bid-158 in Table 2 are consistent with intramolecular distances in Bid. Positions Bid-118, Bid-140, and Bid-158 are within helices α4, α5, and α6, respectively. According to the NMR structure of Bid, the side-chains of these helices are closely packed. It is very likely that the same interactions that pack these helices in soluble, monomeric Bid are maintained in the intermolecular contacts of tBid when it is associated with the OMM in an extended conformation.

### Interprotein contacts between tBid and Bax at the OMM

**Figure 4:**
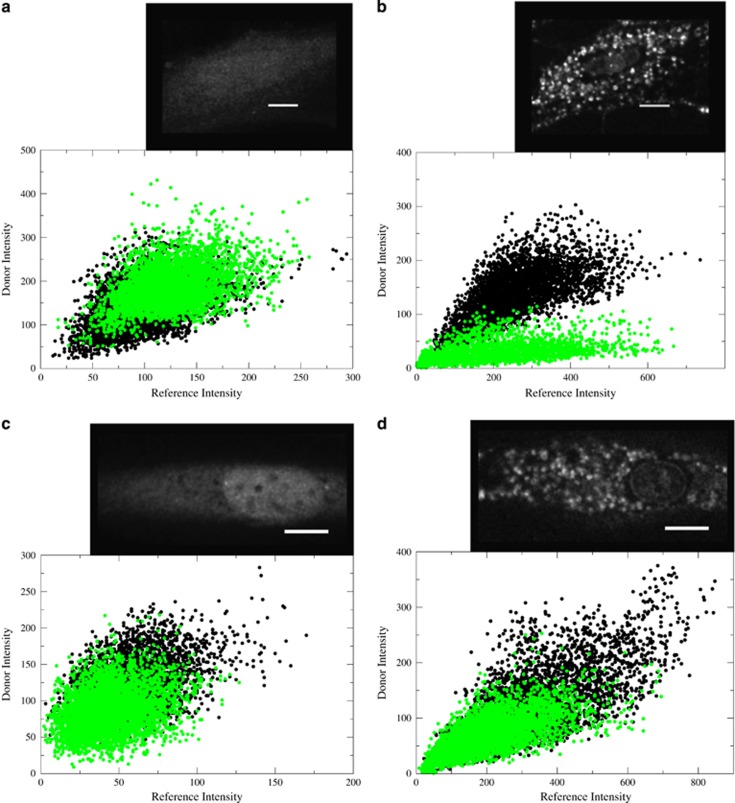
Intermolecular contacts between tBid and Bax molecules. (**a**) Before translocation, there is no FRET between Bid-140 and Bax-78. (**b**) After translocation, FRET is observed between Bid-140 and Bax-78 as evidenced by the lower correlative slope in the presence of the FRET acceptor (green dots) compared with the absence of the acceptor (black dots). (**c**) There is no FRET between Bid-88 and Bax-175 before translocation. (**d**) No FRET is observed between Bid-88 and Bax-175 after translocation as evidenced by the similar correlative slope in the presence (green dots) and absence (black dots) of the FRET acceptor

Contacts between different regions of tBid and Bax at the OMM were also observed. The translocations of Bid-140 and Bid-88 in the presence of Bax variants are shown in Figure 4. As in Figure 3, the intracellular distribution of Bid is diffuse, Figures 4a and c, before becoming punctate after translocation to the mitochondria, Figures 4b and d. In Figure 4a, no FRET efficiency is observed between positions Bid-140 and Bax-78 before translocation as evidenced by the similar correlative slopes in the presence and absence of the FRET acceptor (Dabcyl) conjugated to Bax-78. In Figure 4b, however, after translocation, there is a substantial amount of FRET efficiency between Bid and Bax at these positions as indicated by the significantly lower correlative slope in the scatterplot. In Figures 4c and d, no FRET was observed between positions Bid-88 and Bax-175 as evidenced by similar correlative slopes in the presence and absence of the FRET acceptor at position Bax-175.

Intermolecular FRET efficiencies between tBid and Bax at other positions are shown in Table 3. It is clear from the FRET efficiencies that tBid and Bax interact on the OMM following translocation. Most of the interactions are in the vicinity of the position Bax-78. This position is involved in a dimerization site in the BH3 domain of Bax. To verify that Bax–Bax dimerization remains intact under conditions with an excess amount of microinjected Bid, the intermolecular FRET efficiency was measured between Bax-78 and another site that is involved in this dimerization interface, Bax-62, under similar conditions for microinjection. Using the average of measured FRET efficiency from the images of 12 cells, the intermolecular FRET efficiency between Bax-78 and Bax-62 was 0.46±0.10. This agrees with the previously published value of 0.44±0.12.^22^ When additional Bid was added in equimolar amounts to the two Bax variants (Bax-78 and Bax-62) and microinjected into cells, an intermolecular FRET efficiency of 0.45±0.11 was calculated from measurements of 16 cells. Based on these results the dimer architecture that was previously observed in Bax is intact when excess Bid is also co-microinjected.

## Discussion

Prior to the initiation of apoptosis, Bid adopts a compact conformation similar to the NMR structure of Bax^29^ before associating with the OMM where it adopts an extended conformation that allows it to form intermolecular contacts. This extended structure is consistent with an NMR study, which showed tBid in a micellar environment retained its helical character in an extended conformation.^30^ However, in contrast to Bax, tBid does not have any helices that insert into the membrane. This feature would indicate a weaker membrane interaction of Bid relative to Bax. The weaker membrane interaction may allow it to be more fluid in its association with the OMM and could more easily find another Bax molecule to interact and facilitate the insertion of the membrane-spanning helices in Bax. Prior work has shown that tBid is one of the activators that can induce Bax insertion.^31^ It is also still not fully understood how cleavage at position 59 assists in the translocation of Bid. In fact, Figure 1c shows that translocation can occur without Bid fragmentation into p15 and p7. The analysis of the auto-correlation function of the live-cell fluorescence correlation spectroscopy (FCS) shows that a population of Bid may already interact with the mitochondria, similar to Bax. In addition to the fast time constant, consistent with diffusion of a monomer of Bid, another much longer time constant was measured that is consistent with an oligomerized protein or a protein associated with an organelle. Possibly, factors that can affect the membrane environment in addition to the cleavage are required to allow Bid to associate with the OMM.

Bid and Bax translocate to the mitochondria independent of their relative concentrations. Whether their ratio had excess amounts of Bax relative to endogenous Bid,^22^ equimolar amounts, or excess of Bid relative to endogenous Bax, translocation was observed for both proteins. According to our observations, varying ratios of Bid and Bax also did not alter the rate of translocation to the mitochondria. This is consistent with a previous study^6^ that showed that Bid interacts in a catalytic nature with Bax to aid in the formation of oligomers. Interestingly, the same study also found that Bid may not be a structural element in the complex forming on the mitochondria membrane. A broad range of ratios of Bid and Bax would allow for permeabilization of the OMM under a variety of conditions. In addition, our observations suggest that a bimolecular interaction between Bid and Bax would not be required for insertion to the OMM. These observations are consistent with a recent study by O'Neill and colleagues.^32, 33^ Their genome editing experiments show that Bax and Bak can initiate oligomerization in the absence of any other Bcl-2 protein, including Bid. Interactions with the OMM are sufficient to Bax and Bak to be activated at the initiation of apoptosis. Although they propose that Bid and other BH3-only proteins inhibit the activity of pro-survival proteins, our observations provide another aspect of the activity of Bid where Bid is also involved in the interactions that permeabilize the OMM. By having both proteins independently translocating to the mitochondria, the permeabilization of the OMM could occur more efficiently.

**Figure 5:**
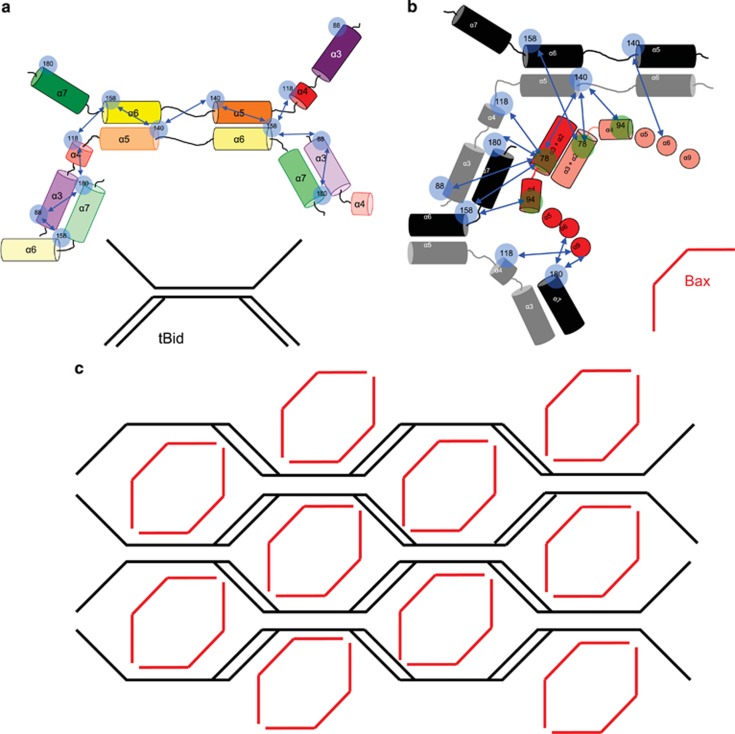
Arrangement of tBid and Bax at the initiation of apoptosis. (**a**) Using the FRET efficiencies in Table 1, a proposed arrangement of tBid helices are shown. Blue circles represent sites where FRET probes were placed on tBid. The helices of tBid are color coded as in Figure 1. (**b**) Using the FRET efficiencies in Table 2, a proposed arrangement of how Bax and tBid contact each other is shown. Alpha helices represented as circles indicate that they are inserted into the mitochondrial membrane, whereas the cylinders show that they are on the outer leaflet. Helices belonging to tBid are in gray and black to distinguish that they are from different tBid monomer, those from different Bax monomers are in red and pink. (**c**) In a simplified notation, an oligomeric pattern of contacts between Bax (red) and tBid (black) is shown

Upon association with the OMM, tBid and Bax form homo- and heteroligomers. Homo-oligomers of tBid (tBid:tBid) have been observed in reconstituted mitochondria^34^ as well as synthetic membrane systems,^18^ whereas in another study, no oligomers were found to be formed.^6, 19^ These seemingly contradictory results highlight the necessity to be able to observe the protein in a specific spatial and temporal manner. Under conditions of live cells undergoing apoptosis, our study is the first to identify where Bax and tBid make specific molecular contact in these oligomers (tBid:Bax). Figures 5a and b show the positions where intermolecular FRET was measured and arrows indicate the observed FRET efficiency. In Figure 5a, none of the efficiencies violate an extended conformation of tBid in the OMM proposed by the intramolecular FRET efficiencies. Although some intermolecular contacts are new, such as between helices α3 and α7, some of the contacts could be preserved from the monomeric form of Bid, such as between helices α4, α5, and α6. Helix α6 from one molecule of tBid could interact with α4 and α5 from another molecule in a similar fashion as exhibited by the NMR solution structure.^5^ Figure 5b shows the observed intermolecular contacts between tBid and Bax. Bax is arranged according to a previous study.^22^ Some of these interprotein contacts may also be preserved from monomeric interactions. The helices α5, α6, and α7 in tBid may interact with BH3 domain in helix α3 of Bax as the structurally homologous helices α4, α5, and α6 in Bax do. Also, Bak is structurally homologous to Bax and, therefore, might interact with tBid in a similar manner. Bax and Bak mixed dimers have been observed.^35^ This potential cross interaction of conserved contacts within the Bcl-2 family could allow for the efficient formation of the Bcl-2 oligomers at the initiation of apoptosis.

A proposed arrangement of interactions between tBid and Bax is shown in Figure 5c. Note that this may not be a unique model that can satisfy our limited number of observed molecular contacts. Even though there are features of this pictorial representation that could have greater precision, it does assist in visualizing the complexity of the tBid and Bax interactions involved in the formation of their oligomers. From our FRET efficiencies it is not clear whether there is a uniform size to the oligomers of these Bcl-2 proteins or a necessary seeding interaction. As mentioned previously, tBid and Bax can translocate and initiate this oligomeric network of interactions independently, which would indicate that these oligomers form at multiple and separate locations on the OMM. In an earlier study^36^ of oligomerization of Bax in liposomes the presence of tBid was increased by increasing the curvature of the liposomes using Drp1. Therefore, if this network of interactions prefers or even promotes membrane curvature, the Bcl-2 oligomers can become self-propogating until the OMM is fully permeabilized. This scenario is reminiscent of proteins that are involved in membrane sensing and remodeling processes in the cell, such as vesicle budding and fusing. In these processes, a number of different cytosolic proteins arrive at the membrane, concentrate into discrete spots, and deform the membrane surface.^37, 38, 39, 40^ Some of the proteins that interact with the membrane contain unique functional features, such as the BAR domain or ALPS motif.^41^ BAR domains exist as monomers in the cytosol.^42^ However, upon recognition of the membrane surface they oligomerize efficiently.^43^ The size of the oligomer assembly is defined by the membrane surface topology. The ALPS motif contains a long amphipathic helix that is composed of bulky hydrophobic residues on one side and a polar side consisting of small and uncharged residues.^44, 45^ ALPS motif can intercalate into lipid-packing defects in a membrane. These defects are the result of the mismatch between membrane curvature and the shape of the lipid molecule.^44, 46, 47^ In comparison, the C-terminal helix α9 of Bax is often referred to as hydrophobic, yet it consists of bulky hydrophobic residues on one side and a polar side composed of glycine (G179), serine (S184), and threonine (T172, T174, T182, and T186). This helix is a crucial component for Bax to target to the mitochondrial membrane. Earlier reports of Bax colocalization with Drp1 and Mfn2^48, 49^ proteins that are involved in mitochondria fission, seem to support that Bcl-2 oligomers can form self-propogating oligomers at discreet regions of the mitochondria.

Direct interaction of Bax with tBid as presented in this work, or with possibly other Bcl-2 members in the creation of protein oligomer networks within the OMM, closely parallels the interaction between proteins that sense and remodel membranes. This analogy also fits well with the concentration independent formation of the Bid-Bax oligomers in the mitochondrial membrane. This feature of the general network of protein interactions on the mitochondrial membrane lends itself to our understanding of the irreversibility of this step in the initiation of apoptosis.

## Materials and Methods

### Production of Bid and Bax mutants

Bid mutants with an N-terminal 6X-Histidine tag were produced by replacing all native cysteines with a serine in the original pET15b-Bid plasmid carrying human Bid cDNA.^30^ This was then followed by the replacement of various residues at desired locations with a cysteine that was used for the conjugation of molecular probes. Bid variants had either one or two cysteines. All mutations were generated using the pET15b-Bid plasmid as a template and the QuickChange site-directed mutagenesis kit (Stratagene, Santa Clara, CA, USA). BL21(DE3) *Escherichia Coli* cells were transformed with the modified plasmids and grown according to previously published conditions,^30^ except Luria Bertani broth was used instead of minimal media with isotopic enrichment. After harvesting and resuspending the cells in lysis buffer (20 mM Tris-HCl, 500 mM NaCl, 5 mM Imidazole), the cells were passed twice through an emulsifier before centrifugation at 34 000 × *g* for 30 min at 4 ^°^C. The cell lysate was then passed through a 5-ml HisTrap HP column (GE Healthcare, Pittsburgh, PA, USA) equilibrated with lysis buffer. The His_6_-tagged Bid variant was eluted using an increasing gradient of elution buffer (20 mM Tris-HCl, 500 mM NaCl, 1 M Imidazole). Bid fractions were then pooled and buffer exchanged into Buffer A (20 mM Tris-HCl, pH=8) using a PD10 column (GE Healthcare). Further purification was performed by anion-exchange chromatography using a 1-ml HiTrap HP Q column (GE Healthcare) equilibrated with Buffer A. Bid was eluted using a linear salt gradient with Buffer B (20 mM Tris-HCl, 1 M NaCl, pH=8). Removal of the Histidine tag was not necessary because His_6_-tagged Bid exhibits wild-type (WT) behavior in live cells (see 'Confocal Imaging for FRET Measurements and Activity Assays'). All Bid and Bax mutants, that are not modified by cross-linking, translocate from the cytosol to the mitochondria at roughly the same rate as the WT proteins.

### Labeling of mutants with fluorescent dyes for FRET measurements

Bid was site-specifically labeled at engineered cysteines using maleimide chemistry. AlexaFluor546 and AlexaFluor488 (Invitrogen) were used as FRET donors and Dabcyl (Anaspec) was used as the FRET acceptor. For the conjugation reaction of each probe to Bid, the conditions were 20 mM Tris-HCl at pH 8.0 with 1 mM TCEP, in the dark for 1 h at 25 ^°^C. The protein concentration was 100 *μ*M. To react the FRET donors to Bid, the ratio of protein to dye was 2:1 for proteins with two cysteines and 1:1 for single-cysteine variants. To conjugate the FRET acceptor, Dabcyl, to Bid a 7:1 excess of dye to protein was used. Unreacted and reacted species with one fluorescent dye molecule were separated with a mono-Q column as described previously.^27^ The internal reference, AlexaFluor633, was conjugated to the above dye-labeled Bid proteins using recently published protocol at Lys144 or Lys146 (Fragment from residues 142–148 had an additional mass corresponding to the AlexaFluor633 tag: *Exp.* 1801.90 Da, *Theor.* 1801.90 Da).^27^ Liquid chromatography mass spectrometry (LC-MS) of the labeled protein variants and their fragments, following pepsin digestion, confirmed the stoichiometry and location of dyes, respectively, as previously described.^27^

### Confocal imaging for FRET measurements and activity assays

The confocal microscopy and microinjection setup to study Bid was described earlier.^22, 27^ All imaging experiments were carried out using serially immortalized mouse embryonic fibroblast (MEF), which was generously provided by Richard Youle, (NINDS, NIH).^48^ Additional experiments were performed on a Zeiss 510 system but with the same excitation and emission filter sets that were used in the previous studies. The total concentration of conjugated protein that was microinjected for FRET measurements ranged from 200 to 400 nM. Equimolar amounts of 200 nM of different Bid variants were co-injected for intermolecular FRET measurements, whereas equimolar amounts of 200 nM of Bid and Bax variants were co-injected for interprotein FRET. To ensure that Bax:Bax contacts were intact for Bid:Bax experiments, an additional 200 nM of unlabeled Bid was added and co-injected with 200 nM of fluorescent Bax labeled at position 78 and 200 nM of Bax labeled at position 62. Previously we estimated that there was a dilution factor of 1:5–1:10 of the injected protein concentration relative to the final concentration in the cell.

FRET efficiency depends on a distance separating two fluorophores. The choice of pair of fluorophores depends on their characteristic Föster radius (R_0_), which corresponds to a distance between the fluorophores with 50% FRET efficiency. To measure intramolecular FRET between two cysteines, the *F'* sample consisted of a Bid variant with two cysteines with only AlexaFluor546 or AlexaFluor488 labeled on one of the cysteines and the internal reference, AlexaFluor633. Then, a *F*_*Q*_*'* sample was prepared for this same variant that was labeled with the either AlexaFluor546 or AlexaFluor488 as well as Dabcyl on the other cysteine in addition to the internal reference, AlexaFluor633. FRET efficiency, *E'* was calculated as described previously.^27^ To measure intermolecular FRET between two cysteines, the *F'* sample was labeled with AlexaFluor546 and the internal reference, AlexaFluor633. The *F*_*Q*_*'* sample consisted of co-microinjecting the *F'* sample with another single-cysteine variant of Bid labeled with Dabcyl. FRET efficiency, *E'* was calculated as described previously.^27^ The locations of the cysteines used in these experiments are indicated in the Tables.

Translocation of Bid to the mitochondria was initiated by the addition of 3.6 *μ*M staurosporin (STS) into the cell culture medium. This method of initiation was chosen to ensure consistency with our previous studies of Bax.^22, 27^ To verify that the translocation of Bid using STS was consistent with inducing translocation of Bid with TNF*α*,^50, 51^ a colocalization assay with Mitotracker Green FM was performed using 100 *μ*g/ml CHX and 1 *μ*g/ml TNF*α* with cells in PBS. Colocalization of Bid and Mitotracker Green FM, as well as similar Bid translocation times, was observed using both methods of apoptosis initiation (Supplementary Figure S1). The caspase inhibitor, Q-VD-OPh (Biovision, Milpitas, CA, USA) was utilized to examine the effects of caspase-8 induced fragmentation. For these experiments, cellular media was incubated for 15 min of 10 *μ*M Q-VD-OPh before microinjection and STS-induced apoptosis. Experiments utilized 10–15 cells to determine the FRET efficiency between two locations. Quantification of fluorescence intensities and further analysis of confocal images were described previously.^22, 27^

### FCS measurements on bid and cross-linked variants

FCS was utilized to measure the intracellular diffusion of Bid and its variants before and after translocation, by counting the number of fluorescence molecules going into and out of the focal volume of the microscope. FCS auto-correlation curves were acquired on the Zeiss 510 system.^52^ Rhodamine 6G was used to determined fitting parameters for a 3-D diffusion model.^52^ Fluorescence signal was acquired as a function of time at three observation locations on each of three cells. The power of the 633 nm laser used in this experiment was <1 mW. The auto-correlation function from each of these locations was normalized and averaged for each sample. For each location, data were collected for two successive 60-second periods. IGOR software was used to determine the time constants for diffusive behaviors as well as their relative populations.

To verify whether the observed conformational changes had an effect on translocation, two variants of Bid were cross-linked with BM(PEG)3 (ThermoFisher, Waltham, MA, USA). One variant was cross-linked between Arg88Cys and Lys158Cys, whereas the other variant was cross-linked between Gln136Cys and Gln180Cys. The conditions for the cross-linking were 35–60 *μ*M Bid, 100 *μ*M BM(PEG)3, and 1 mM TCEP in 100 mM Tris-HCl at pH 7.5 for 4 h in the dark at 37 ^°^C. Completeness of the reaction was verified by LC-MS via the addition of 352.13 Da to the mass of the Bid variant (between Gln136Cys and Gln180Cys: exp. 23130.6 Da, *theor.23130.4* Da; between Arg88Cys and Lys158Cys: exp. 23135.5 Da, *theor. 23136.1* Da). Fragments produced by pepsin that are linked by the cross-linker were also detected (For Bid-88-158, between fragments 154–158 and 83–88: exp. 1234.69 Da, *theor. 1234.69* Da; between fragments 154–165 and 82-88: exp. 2027.10 Da, *theor. 2027.10* Da. For Bid-136-180, between fragments 136–143 and 178–182, exp. 1558.66 Da, *theor. 1558.66* Da; between fragments 135–141 and 178–184: exp. 1668.81 Da, *theor. 1668.81* Da; between fragments 136–143 and 178–184: exp. 1815.80 Da, *theor. 1815.81* Da; between fragments 135–143 and 178–184: exp. 1928.89 Da, *theor. 1928.89* Da). Subsequent to cross-linking, these variants were labeled with AlexaFluor633 and microinjected into MEF cells for analysis.

## Figures and Tables

**Table 1:** Intramolecular FRET efficiencies measured in Bid before and after translocation, preSTS and postSTS, respectively

	**preSTS**	**postSTS**
*Bid-136-180*		
*F'*	1.65±0.11 (6 cells)	0.40±0.10 (6 cells)
*F*_*Q*_*'*	1.11±0.23 (6 cells)	0.40±0.08 (6 cells)
*E'*	0.33±0.14	*NF*^a^
		
*Bid-118-180*		
*F'*	1.65±0.33 (5 cells)	0.71±0.05 (5 cells)
*F*_*Q*_*'*	0.43±0.17 (5 cells)	0.36±0.02 (5 cells)
*E'*	0.74±0.12	0.49±0.05

a *NF* indicates there is no statistical difference between the *F'* and *F*_*Q*_*'* values, which corresponds to no FRET efficiency between the two locations

**Table 2:** Intermolecular FRET efficiencies measured between tBid molecules after translocation

**Residue**	**Bid-118**	**Bid-140**	**Bid-158**	**Bid-180**
Bid-88	*NF*^a^	*NF*	0.51±0.13	0.44±0.11
Bid-118	*NF*	*NF*	0.54±0.12	0.36±0.14
Bid-140	-	0.20±0.16	0.58±0.10	*NF*
Bid-158	-	-	*NF*	*NF*
Bid-180	-	-	-	*NF*

a *NF* indicates there is no statistical difference between the *F'* and *F*_*Q*_*'* values, which corresponds to no FRET efficiency between the two locations

**Table 3:** Intermolecular FRET Efficiencies measured between tBid and Bax molecules after translocation

**Residue**	**Bax-78**	**Bax-94**	**Bax-145**	**Bax-175**
Bid-88	0.32±0.18	*NF* a	*NF*	*NF*
Bid-118	0.50±0.14	*NF*	*NF*	0.54±0.12
Bid-140	0.70±0.09	0.20±0.18	0.30±0.27	0.38±0.15
Bid-158	0.52±0.18	0.33±0.17	*NF*	*NF*
Bid-180	0.43±0.13	*NF*	0.32±0.27	0.24±0.18

a *NF* indicates there is no statistical difference between the *F'* and *F*_*Q*_*'* values, which corresponds to no FRET efficiency between the two locations.

## Abbreviations

Bcl-2: B-cell lymphoma 2
BH1-4: Bcl-2 homology domains 1–4
CHX: cycloheximide
FCS: fluorescence correlation spectroscopy
FRET: Förster resonance energy transfer
LC-MS: liquid chromatography mass spectroscopy
MEF: mouse embryonic fibroblasts
MOM: mitochondrial outer membrane
MOMP: mitochondrial outer membrane permeabilization
NMR: nuclear magnetic resonance
OMM: outer-mitochondrial membrane
PBS: phosphate-buffered saline
Q-VD-OPh: (3S)-5-(2,6-Difluorophenoxy)-3-[[(2S)-3-methyl-1-oxo-2-[(2-quinolinylcarbonyl)amino]butyl]amino]-4-oxo-pentanoic acid hydrate
STS: staurosporin
TNF*α*: tumor nercrosis factor *α*
Tris-HCl: Tris(hydroxymethyl)aminomethane hydrochloride
WT: wild-type
