# Supplementary material for: Bcl-2 proteins bid and bax form a network to permeabilize the mitochondria at the onset of apoptosis
Source: Cell Death Dis. 2016 Oct 20;7(10):e2424–. doi: 10.1038/cddis.2016.320 (PMC5133987; doi:10.1038/cddis.2016.320)
Supplement: Supplementary Material [file cddis2016320x1.docx]

**SUPPLEMENTARY INFORMATION**

**Bcl-2 proteins Bid and Bax form a network to permeabilize the mitochondria at the onset of apoptosis**

Robert F. Gahl, Pallavi Dwivedi and ^1^Nico Tjandra

Laboratory of Molecular Biophysics, Biochemistry and Biophysics Center, National Heart, Lung and Blood Institute, National Institutes of Health,

Bethesda, MD 20892, USA.

^1^ Corresponding author:

Nico Tjandra, Ph.D.

50 South Drive, Building 50 Room 3503

Bethesda, MD 20892-8013, USA

Phone: (301) 402-3029

Fax: (301) 402-3405

Email: [tjandran@nhlbi.nih.gov](mailto:tjandran@nhlbi.nih.gov)

*Conformational Changes in Bid resulting from translocation to the mitochondria*

The conformational changes in Bid were probed by measuring the distance from Gln180Cys to two separate locations: Arg118Cys and Gln136Cys, Figure 1A&B, by observing changes in FRET efficiency, Table 1. The FRET efficiency was calculated using the intensity of the FRET donor relative to the internal reference in the presence (*F_Q_´*) and absence (*F´*) of the FRET acceptor as previously described (22,27 in the main text). AlexaFluor488 and Dabcyl with an R_0_ of 50 Å were used to probe conformations between Bid-118 and Bid-180, while AlexaFluor546 and Dabcyl with an R_0_ of 29 Å were used between Bid-136 and Bid-180.

FRET efficiencies, measured between positions Bid-118 and Bid-180, as well as positions Bid-136 and Bid-180, in a cuvette were 0.42 and 0.63, respectively. Distances measured in the NMR structure between Cα atoms of Bid-118 to Bid-180 is 17.3 Å and of Bid-136 and Bid-180 is 19.8 Å (PDB ID: 2BID)(ref. 5 in the main text). The efficiency for Bid-136 and Bid-180 corresponds to a distance consistent with Bid being in a compact conformation similar to its structure, while efficiency for Bid-118 and Bid-180 suggests they are further apart than indicated in the structure. To verify that the mutations at various locations within Bid did not affect its native structure, circular dichroism (CD) spectra, Figure S2, were acquired for each mutant. The CD spectrum of each mutant shows that the secondary structure is still similar to WT Bid. The FRET efficiencies between the same pairs were also measured in the cell before Bid translocation, and they were 0.74 ± 0.12 and 0.33 ± 0.14 for positions Bid-118 and Bid-180 and Bid-136 and Bid-180, respectively. The differences between the in-cell and cuvette measurements can be explained by the presence of a small population of Bid that is associated with the mitochondria membrane prior to apoptosis induction. Note, the effective FRET efficiency is just an average of the equilibrium population of cytosolic Bid (with FRET efficiencies equal to the values measured in the cuvette) and the membrane associated Bid. Taking into account the FRET efficiencies of Bid after translocation, the FRET efficiency between positions Bid-136 and Bid-180 is expected to decrease compared to the cuvette data. Interestingly, Bid-118 and Bid-180 pair FRET efficiency is more in line with what’s expected. Considering that their distance in the structure is shorter than the Bid-136 and Bid-180 pair, and the R_0_ for Bid-118 and Bid-180 fluorophores is 50 Å, compared to 29 Å for the other pair, one can expect FRET efficiency around 80%. Additionally, taking into account the possible contribution form membrane associated population then the observed FRET efficiency for this pair is within expected range.

After translocation, tBid rearranges its conformation and is intimately associated with the membrane. For tBid, there is no FRET efficiency between positions Bid-136 and Bid-180, which indicates that these residues are at least 45 Å away from each other. Bid, when in an extended conformation yet with helices intact satisfies this distance restraint. Interestingly, FRET efficiency is detected between positions Bid-118 and Bid-180 after translocation. However, as will be described in the section, “*Intermolecular Contacts between tBid* *molecules at the OMM*”, there is an intermolecular component to the observed FRET efficiency. When this contribution is subtracted from the observed FRET efficiency, the remaining contribution of intramolecular FRET is negligible. Therefore, the lack of FRET efficiency between positions Bid-118 and Bid-180 is consistent with the observations between positions Bid-136 and Bid-180 and confirms that tBid adopts an extended conformation yet have its helices intact. This is consistent with tBid NMR structure that was determined in a detergent micelle (ref. 26 in the main text). In this structure (PDB ID:2M5I) measured distances between the Cα atoms of Bid-118 and Bid180 is 61.3 Å, while between Bid-136 and Bid-180 is 35.6 Å.

**Figure S1**. *Co-localization of Bid and mitochondria after translocation initiated by STS and TNFα* A)Microinjected Bid undergoes translocation after the addition of STS to the cellular media. The cellular distribution of Bid goes from diffuse (preSTS) to punctate (postSTS) and co-localizes with MitoTracker Green (postSTS, Overlay). B)The same transition and co-localization is observed when translocation is initiated by CHX and TNFα.

**Figure S2**. *Intact secondary structure after various mutations in Bid.* The secondary structure for each of the Bid mutations was measured by circular dichroism (CD) spectroscopy. The secondary structure for each mutant was intact.

**Figure S1**

**Figure S2**
